# Using a Transdisciplinary Interpretive Lens to Broaden Reflections on Alleviating Poverty and Promoting Decent Work

**DOI:** 10.3389/fpsyg.2016.00503

**Published:** 2016-04-12

**Authors:** Annamaria Di Fabio, Jacobus G. Maree

**Affiliations:** ^1^Department of Education and Psychology (Psychology Section), University of FlorenceFlorence, Italy; ^2^Department of Educational Psychology, University of PretoriaPretoria, South Africa

**Keywords:** decent work, poverty, transdisciplinary perspective, intervention, fair and sustainable development, new responsibility for decent work agenda

## Abstract

This article aims to broaden current reflections on definitions of decent work and poverty using a transdisciplinary interpretive lens comprising philosophical, juridical, economic, sociological, and psychological understandings. We (the authors) undertook an adapted systematic qualitative review to gather data on different perspectives on decent work and poverty. The article summarizes and compares reflections on the two constructs and proposes an enhancement of the current definition of decent work. The aim is to facilitate the identification and development of new research and intervention projects that can be implemented to promote fair and sustainable economic development, the provision of decent work, and the reduction of poverty globally. We believe that challenges should be dealt with pro-actively rather than reactively and that intervening at the level of primary prevention should lie at the heart of any strategy to promote decent work and alleviate poverty. Radical intervention is needed to ensure that future generations not only survive but develop, grow, and express themselves meaningfully through decent work

## Introduction

Somavia (1999, n.p.) describes decent work as “productive work in which rights are protected, which generates an adequate income with adequate social protection…and employment, income and social protection can be achieved without compromising workers’ rights and sound standards.” The author maintains that decent work deficit occurs when:

• involuntary unemployment and poverty are evident;
• abuses of rights occur in the workplace and when forced child labor exists;
• basic income security is compromised;
• employees and employers are not organized well enough to express themselves openly or when effective dialogue is impeded; and when
• work-life is not balanced with legitimate family demands.

According to the United Nations [UN] (2006), “The Decent Work Agenda promotes access for all to freely chosen employment, the recognition of fundamental rights at work, an income to enable people to meet their basic economic, social and family needs and responsibilities and an adequate level of social protection for workers and their family members” (p. 85). This definition (adopted by the International Labour Organization [ILO], 1999) was interpreted by the international community as productive work for male and female employees in work environments that promote freedom, equity, security, and human dignity. Decent work, defined thus, encompasses jobs that (a) deliver a fair income, (b) are characterized by workplace security, (c) provide protection for workers and their families, (d) offer opportunities for personal development, (e) encourage social integration, (f) allow workers to express their concerns, (g) allow workers to organize themselves into interest groups that can contribute to decisions that influence their lives, and (h) guarantee equal opportunities and equal treatment to everybody. The Decent Work Agenda thus reflects a balanced and integrated approach to the pursuance of broad and productive employment and decent work for all workers at international, national, regional, sector-specific, and local levels [FAQs/Useful definitions (UN sources), n.d., n. p.].

The aims of the Decent Work Agenda are in line with the UN’s 17 sustainable development goals (Sustainable development goals, 2015). These 17 goals confirm the importance of promoting decent work for all as such work enhances opportunities for progress, counters marginalization, and helps develop individuals, families, communities, and countries (in short, promotes sustainable development and growth globally) (Guichard, 2013; Di Fabio, 2015; Guichard and Di Fabio, 2015).

### Aims of the Study

The study discussed here was a response to calls (Guichard, 2013; Di Fabio, 2015; Guichard and Di Fabio, 2015) for further reflections and research on the definition of decent work and poverty. The study aimed to deepen our understanding of decent work and poverty using a transdisciplinary interpretive lens comprising philosophical, juridical, economic, sociological, and psychological perspectives on the two constructs (decent work and poverty). We attempted to identify similarities as well as differences between the constructs. More particularly, we endeavored to harness transdisciplinary perspectives, theories, and definitions in order to gain some insight into critical global humanitarian needs. It is hoped that this will cast new light on current and envisaged research and interventions aimed at promoting decent work and reducing poverty thereby contributing to the realization of the aims and goals articulated by the UN and the ILO.

### Working Assumptions

The study was premised on the belief that decent work can be provided and that poverty can be ended. However, interventions at the level of tertiary prevention of poverty or even at the second level of prevention only are not sufficient. Interventions at the level of primary prevention should override all other considerations and forms of intervention (Hage et al., 2007; Kenny and Hage, 2009).

### Research Method

An adapted version of the systematic qualitative review (recommended by Higgens and Green, 2011) and others was undertaken of the literature on decent work and poverty. Given that this is an entirely qualitative study, and mindful of the words of Booth (2001, p. 1), namely “Why should systematic reviewers of qualitative research pursue a ‘gold standard’ comprehensive literature search when concepts such as ‘data saturation’ have an established pedigree?”, we purposefully chose to rather search for sources until we believed that no new ‘themes’ emerged (in other words, until we had reached a point of data saturation). The review focused on scholarly articles and books and included also older, seminal sources. Data were collected by examining various studies, and the findings were synthesized. The following procedure was followed:

(i) We requested academic information specialists to search for abstracts of a number of databases for information (international books, articles, reference works, conference papers but also other relevant publications) on our topic. These specialists first chose platforms and databases; EBSCOHOST being the basic platform. EBSCOHOST Databases included: Academic Search complete, Masterfile, Eric, PsycInfo, PsycArticles, and Sociological Abstracts.
(ii) We did a simultaneous search (Google and Google Scholar) to improve or chances of identifying the widest range of sources possible.
(iii) Duplicate sources were removed.
(iv) Once we had received the information from our academic information specialists, we examined sources appropriateness and subsequently requested full-texts.

Inclusion criteria included the following: sources were included if we believed that sufficient ‘evidence’ were provided that they reflected contributed to elucidation of the topic, if they reflected more than mere personal opinions, and if they were written in English and Italian. No specific data range was specified. Dated sources believed to be sufficiently seminal were thus also included.

Exclusion criteria included the following: sources were included if we believed that insufficient ‘evidence’ was provided that they reflected more than mere personal opinions, if we believed that they were not meaningfully related to our topic, and if we believed that they reflected and form of bias. Dated sources believed to be insufficiently seminal were thus excluded.

### Data Analysis

Braun and Clarke’s (2006) thematic analysis was adapted to identify, analyze, and report themes (perspectives) and subthemes (subperspectives) that emerged. More specifically, we made a conscious, inductive attempt to determine a number of recognizable perspectives on our topic. We familiarized ourselves with the large number of sources, read and re-read them, after which we generated preliminary codes systematically. Next, we organized the codes into five themes (perspectives) and subthemes (sub-perspectives), which were subsequently reviewed labeled. We concluded by writing our manuscript.

Below, we provide substantiation for the five perspectives and make it clear to the reader how specific studies contributed to certain perspective on decent work and poverty.

## Perspectives on Decent Work

### The Philosophical Perspective on Decent Work

The philosophical approach to decent work centers on dignity as a fundamental dimension of decent work (Peruzzi, 2015) and holds that the notion of dignity varies within and between cultures. This is consistent with perspectives that challenge the assumption of universal human rights and maintain that any particular doctrine is always relative and not universally shared. The assumptions underlying the slogan that “all men [sic] are equal” that preceded the *Universal Declaration of Human Rights* (UDHR) and its reframed, politically more correct, slogan “all human beings are born free and equal in dignity and right” (United Nations, 1948, General Assembly) are clearly incorrect. Human beings are ideally considered equal in terms of their rights, yet, in reality, they do not have the same opportunities. Peruzzi (2015) believes that the assumption of equality leads to a rethink of concepts such as rights and dignity: if human beings have an inalienable claim to equal rights, they also have the right to be different to other people (to lead different lives and choose and execute different occupations). Furthermore, decent work should not be considered solely from a “Western” or “Eurocentric” point of view.

Seen from a human rights perspective, decent work intrinsically has four dimensions (Peruzzi, 2015): freedom (to choose or refuse a specific type of work); equity (to have a fair income); security (including something as basic as access to primary health care); and dignity. If the notion of a decent society implies a society that is fair and just (Arneson, 2007), then it follows that organizational and occupational contexts and work lives should also be equally fair and just (decent). The idea of respecting a person’s honor and reputation (Article 12 of the UDHR) and the entitlement to the unrestricted development of one’s own personality (Article 22) lie at the heart of the notion of decent work. This underlies the basic right to be protected against humiliation and dehumanization. The same applies in occupational contexts where workers are not free to choose what work they wish to do and, instead, have to accept mandatory work. The opposite of mandatory work, namely decent work, is promoted when working environments are structured to enhance dignity, respect, humane working conditions, and adequate self-construction (Guichard, 2009). What is needed is the active promotion of such environments and the identification of contexts where action is needed (Peruzzi, 2015). Examples of such contexts are those where delocalization in manufacturing services and the extractive industry (Peruzzi, 2015) occur. This boils down to the importance of changing economic models rather than attempting to perpetuate the myth of promoting economic growth to enhance sustainable development. Suffice it to say that the idiosyncratic logic (revealed by this myth) entertained by many global economies is easily shown up when analyzed through a humane and visionary analytic lens.

### The Juridical Perspective on Decent Work

The current kaleidoscopic assortment of juridical definitions of decent work has at one end of the range Article 23 of the United Nations (1948) Universal Declaration of Human Rights:

“(1) Everyone has the right to work, to free choice of employment, to just and favorable conditions of work and to protection against unemployment.
(2) Everyone, without any discrimination, has the right to equal pay for equal work.
(3) Everyone who works has the right to just and favorable remuneration ensuring for himself (sic) and his family an existence worthy of human dignity, and supplemented, if necessary, by other means of social protection.
(4) Everyone has the right to form and to join trade unions for the protection of his interests.”

At the other end of the range, Faioli (2009) distinguishes between decency at work and dignity at or in work. Decency at work implies the pre-legal assumption of modernizing labor law and is defined in terms of the application of social security, which is regarded as an indispensable ingredient of decent work. Dignity at or in work refers to the legal assumption of decency at work and is associated with equal opportunities and workers’ entitlement to social security rights. Dignity in work concerns work quality and implies that workers absorbed or involved in their work should be allowed to combine their lives (in terms of their work-related vision and their personal life) with their work (Faioli, 2009).

International consensus exists on the need for labor law to promote decent work at national level in all countries. High stakes are involved because the labor law system is expected to restore and maintain equilibrium in the labor market in terms of (a) facilitating access to new employment opportunities and (b) reinforcing employability policies (Faioli, 2009). Promoting employment opportunities and workers’ employability have lately emerged as critical ingredients of decent work.

### The Economic Perspective on Decent Work

The United Nations Economic and Social Council (2006, n.p.), by means of a General Comment, defined decent work in terms of meeting the minimum requirements stipulated in Article 7 of the International Covenant on Economic, Social and Cultural Rights: “The right of everyone to the enjoyment of just and favorable conditions of work.” More particularly, reference is made to “(a) remuneration which provides all workers, as a minimum, with: (i) fair wages and equal remuneration for work of equal value without distinction of any kind, in particular women being guaranteed conditions of work not inferior to those enjoyed by men, with equal pay for equal work; (ii) a decent living for themselves and their families; (b) safe and healthy working conditions; (c) equal opportunity for everyone to be promoted in his/her employment to an appropriate higher level, subject to no considerations other than those of seniority and competence; (d) rest, leisure and reasonable limitation of working hours and periodic holidays with pay, as well as remuneration for public holidays.”

Moreover, as regards an economic perspective on decent work, Lavagnini and Mennella (2015, pp. 5–11) introduced a multidimensional concept of decent work that includes three kinds of profiles based on different indicators. First, a basic profile with indicators corresponding to daily working conditions. Second, a fairness profile with indicators corresponding to how people with different kinds of personal characteristics should be able to access the same job. Third, a relations profile with indicators involving the social aspect of working. Indicators in the basic profile include working hours (or time actually spent on work activities), physical safety, legality of jobs, and unemployment rate pressure. Indicators in the fairness profile include the irrelevance of private characteristics (e.g., gender, religion, nationality) and hanging work (including lengthy non-productive working conditions and social protection such as unemployment benefits). Indicators in the relations profile include monetary indicators (financial sufficiency) and monetary as well as educational enrichment (the percentage of workers who received training during the last 12 months in a certain job) as well as freedom to voice grievances and to embark on strikes.

An overview of the economic perspective on decent work reveals that its main aim is to detect “labor unfreedoms” (Sen, 1999, p. 3), implying that overcoming these constraints will enhance realization of the wide range of human capabilities and promote human development (Sen, 1999). Seen from this perspective, decent work is linked closely to the attainment of a minimum standard of living (Stiglitz et al., 2010) and becomes a key determinant of quality of life, especially in economic terms.

### The Sociological Perspective on Decent Work

Sociological perspectives on decent work relate mainly to progressive definitions of the term by the ILO. Decent work was initially defined as “productive work, in conditions of freedom, equality, security and human dignity” (International Labour Organization [ILO], 1999, n.p.). More recently, the definition was changed to “opportunities for work that is productive (to satisfy individual basic needs and to give a contribution to society) and delivers a fair income, security in the workplace and social protection for families, better prospects for personal development and social integration, freedom for people to express their concerns, organize and participate in the decisions that affect their lives and equality of opportunity and treatment for all women and men” (International Labour Organization [ILO], 2015, n.p.). The initial International Labour Organization [ILO] (1999) definition of decent work included the following four pillars of decent work: “(1) Workers’ rights, the ethic and juridical basis of decent work. (2) Employment, vital element of decent work (productive work, freely chosen and adequately remunerated for everyone). (3) Social protection, promoting the well-being and social inclusion of the more vulnerable people in the labor market (worker protection). (4) Social dialog, involvement of the workers and participation through their representatives; participatory democracy (voice of workers).” These four pillars have evolved into the following four strategic objectives advocated in the current International Labour Organization [ILO] (2015, n.p.) definition of decent work: “(1) Promoting jobs; … (2) Guaranteeing rights at work; … (3) Extending social protection, to promote both inclusion and productivity; … [and] (4) Promoting social dialog.”

Sociologically oriented reflections on decent work draw on both ILO definitions (International Labour Organization [ILO], 1999, 2015) and hinge on the following themes: Work has to be safe and secure, has to provide a livable income (which means that no person’s income should fall below the minimum income needed to ensure a reasonable living, health, and dignity), has to recognize workers’ basic rights (including the absence of discrimination or harassment), has to ensure that income earned enables workers to meet their basic economic, social and family needs and responsibilities, has to ensure that work offers social protection for workers and their family members, has to ensure that work allows freedom of speech in the work context and representation at work through self-chosen representatives, and has to ensure that work promotes health and lower levels of disease and injury (International Labour Organization [ILO], 1999, 2015). UNESCO’s (2009) definition sheds light on the sociological perspective of decent work. It supports the right to decent work by advocating the provision of adequate work of acceptable quality (protecting workers’ rights and ensuring that workers generate an adequate income), the advancement of diverse income-generating pursuits (salary employment, self-employment, and working from home), fair and favorable conditions for income generation (adequate wages, occupational safety and health, hours of work, and the right of workers to organize), and work that promotes the dignity of the person.

### The Psychological Perspective on Decent Work

Psychology of Working Theory (PWT, Blustein, 2006) emphasizes the importance of satisfying workers’ needs for power, relationships, and self-determination because they involve activities that are experienced as authentic and motivating.

The humanitarian work psychology perspective (Carr et al., 2013) concerns ensuring decent work for all workers that entails challenging responsibilities and offers opportunities for promotion. The aim is to prevent marginalization of workers by granting them access to valued tasks, reducing their work stress and chronic work overload, recognizing their achievements, and facilitating their access to a living wage and work-related support structures. Many people in developing countries in particular work in the informal economy, which is often characterized by unsafe work conditions and low pay. In terms of this perspective, the fight against poverty is advanced by promoting the ideals of economic growth, equitable and sustained employment opportunities, and quality of work, especially among the poor (Bell and Newitt, 2010).

Burchell et al. (2014) reflect on decent work in terms of job satisfaction, intrinsic job quality, and a job desirability index. Job satisfaction refers to the relationship between workers’ perceptions of the quality of work and their expectations of work (Agassi, 1982). The writers also consider workers’ ability to adapt to adverse conditions (Nussbaum, 2004). Intrinsic job quality refers to workers’ well-being and employers’ contribution to it through health schemes, education, compensation schemes, child care programs, adequate wages, and promotion opportunities (Burchell et al., 2014). Index of job desirability refers to objective job characteristics that can promote quality of work and workers’ appraisal of their situation and contribution (Burchell et al., 2014).

Ferrari (2009) broadens psychological perspectives by stressing the value of (any) work as an instrument in combating poverty. She also highlights the importance of the right to work and workers’ rights as well as the meaning of work in terms of its power to facilitate cohesion (work promotes socialization) and its instrumental value (work facilitates access to goods). Ferrari (2009) reflects on (a) employment in terms of quality, quantity, and employment as seen from a decent work perspective, (b) underemployment in terms of challenges but also as “opportunities to organize personal alternative spaces” (Ferrari, 2009, p. 7), and (c) challenges for research in psychology to shed light on what dignity entails by also focusing on the extent to which dignity is afforded to workers in non-traditional occupational contexts. Ferrari’s views are corroborated by those of many other scholars (Wilson, 1996; Blustein, 2008; Paul and Moser, 2009; Swanson, 2012; Wanberg, 2012; Guichard, 2013), who highlight the strong, positive relationship between decent work (which affords workers dignity) and workers’ well-being. Put differently: access to decent work is a prerequisite for the psychological health of individuals and groups (Blustein, 2008; Paul and Moser, 2009).

Career psychology, too, advocates the promotion of decent work. The 2001 International Association for Educational and Vocational Guidance (IAEVG) declaration states: “Effective educational and vocational guidance and counseling can assist individuals to understand their talents and potential and enable them to plan the appropriate steps to develop essential skills that will lead to personal, educational, economic and social advancement for the individual, family, community and nation” (The International Association for Educational and Vocational Guidance [IAEVG], 2001, n.p.). Athanasou (2010) stresses the link between decent work and career by arguing that the notion of decent work is recognized and promoted globally and includes reasonable income, equal opportunities, safe working conditions, trade union representation, and a social safety net. This view underlines vocational counselors’ social obligation to advance equity and fairness in occupational contexts.

All this confirms the challenge faced by career counseling and guidance to (a) conduct in-depth global research on decent work, (b) promote the implementation of decent work globally and thereby advance fair and sustainable development, and (c) move beyond and expand the ILO definition of decent work (Guichard, 2013). Serious consideration should be given to the question as to whether work (as defined by the ILO) can be regarded as “decent” if it proves to be damaging to human life or the environment (Guichard, 2013).

Based on the Psychology-of-Work Framework (PWF) (Blustein, 2006, 2013), Duffy et al. (2016) recently proposed a conceptualization of decent work that incorporates contextual factors into an index of one’s own psychological experience. Duffy et al. (2016) also underline the fact that decent work is embedded in self-determination, which in turn leads to meaningful and fulfilling work.

In summary: It is clear that the definition of decent work is still open to discussion and debate. Further in-depth reflection and research are needed to arrive at a generally agreed upon definition (Guichard, 2013; Guichard and Di Fabio, 2015).

## Perspectives on Poverty

### The Philosophical Perspective on Poverty

The distinction between absolute and relative poverty lies at the root of the philosophical perspective on poverty (Peruzzi, 2015). Whereas absolute poverty refers to biological deprivation in terms of the daily minimum intake of food and water, relative poverty refers to the maintenance of a preferred standard of living that changes in accordance with societal or historically determined norms and standards. Many parts of Africa and Asia display the typical characteristics of absolute poverty. Peruzzi (2015) states that emergency help is often provided in circumstances of absolute poverty by external entities, but, as crucial as it is, this kind of intervention does not resolve the challenge of absolute poverty. Absolutely poor refugees, for instance, often escape from war and famine and are subsequently helped to meet their immediate needs – only to end up experiencing extreme relative poverty. This highlights the need to implement medium- and long-term projects to help (absolutely) ‘poor people’ develop and grow instead of merely survive. Narveson (2004, p. 347) argues that “the road to poverty is [often] paved with bad economics.” No one single strategy or approach will work for all victims of poverty. Strategies should be devised to help people realize their full potential. A philanthropic approach alone will not help as feeling sorry for the poor can never be regarded as a long-term solution for poverty. What is needed is a defensible strategy, based on sound business practices, to help poor people earn a decent wage (Narveson, 2004). Education and knowledge acquisition, as the cornerstone requirements for any democracy, will promote a stable income and advance people’s well-being (Peruzzi, 2009).

Cognizant of what constitutes a globally acceptable standard of living, more people will want to purchase goods that will help them meet that standard, and they will therefore need to work harder in order to earn more money, thereby stimulating economic activity. Failure to earn the money required to buy basic goods implies poverty and a lack of well-being. People who are affected often become despondent, fail to find employment, and are eventually denied access to a living wage. Failure to achieve the global standard of living means that people will either remain spectators and passively accept being poor and unhappy, or they will protest against laws they regard as condemning them to a life of poverty. The two main risks here are (a) the risk of unhappiness, and (b) the risk of promoting crime, both of which run contrary to basic ethical principles (Peruzzi, 2015).

Lastly, Peruzzi (2015) considers the challenge of poverty from a universal point of view taking all cultures into account, not just Western culture. This will facilitate more encompassing, culturally based interventions to reduce absolute and relative poverty.

### The Juridical Perspective on Poverty

From a juridical perspective, poverty is a human condition characterized by deprivation in terms of the means to survive and make a reasonable living (United Nations [UN], 2003). The Despouy (1996, p. 2 and p. 13) (Part III) lists the following juridical criteria for poverty: “The denial of human rights as a whole […] the extent to which poverty is a violation of economic and social rights, of civil, political and cultural rights, of the right to development”; “an accumulation of mutually reinforcing misfortunes: poor living conditions, insalubrious housing, unemployment, ill health, lack of education, marginalization, etc., a veritable ‘horizontal vicious circle’ of poverty”; “deprivation of one right can have repercussions on the exercise of the rest”; “tendency of the phenomenon to perpetuate itself by being passed on from one generation to the next: ‘vertical vicious circle’ of poverty”; “social consequence of poverty: exclusion and stigmatization of the poor.”

These criteria make it possible to draw a “juridical” distinction between absolute poverty and relative poverty (Osia, 2010). While absolute poverty is considered as a state of being that deprives individuals of the means to meet their basic needs (Kokaz, 2007), relative poverty refers to comparative societal deprivation and different expectations of justice (Brandt, 1980). Burton (2007) lists the following criteria for assessing poverty: dearth, deficiency, indigence, paucity, and deprivation.

There are juridical indices of poverty such as the Human Poverty Index (HPI) and the Human Development Index (HDI) (United Nations Development Program [UNDP], 2004). Whereas the HPI is a summary measure of deprivation in terms of outcome indicators for identifying unmet basic needs, the HDI reflects the average achievements of countries in terms of citizens’ ability to lead a long and healthy life, to be well educated, and to have a decent standard of living. Poverty is thus an outcome of economic processes and of interacting economic, social, and political forces.

### The Economic Perspective on Poverty

From an economic perspective, poverty exists when income levels do not permit the satisfaction of basic needs. A distinction can be drawn between absolute and relative poverty (Dizionario di Economia e Finanza, 2012; Treccani Etymological Dictionary, 2012): absolute poverty is defined as the lack of the money needed at a given moment to purchase a basket of essential goods and services to just exist. Relative poverty refers to inequality and the income differences between diverse social groups. The International Standard of Poverty Line (ISPL) (Treccani Etymological Dictionary, 2012) is used to measure poverty (relative to a given population’s average living standard). The Treccani Etymological Dictionary (2012) points out that the word “poverty” stems from the Latin words “parere” (to obtain or produce) and “pauper” (a person who produces little, has inadequate economic resources, and is therefore unable to support himself/herself).

A review of the evolution of the definitions of poverty over the past few years casts light on the economic perspective on the topic. For example, a distinction was drawn in the 19th century between groups referred to as “pauvre” and “indigent” (Hulme and McKay, 2005). While the pauvre experienced cyclical poverty when crops failed or when the demand for seasonal agricultural labor was low, the indigent were perpetually poor due to of ill health, accidents, old age, or intoxication. In the 20th century, the focus in definitions shifted to the persistence of poverty (at individual and household levels) as well as the high, positive correlation with poverty and the serious effects of poverty (Gaiha, 1993). Attention was also paid to the complexity of the phenomenon, the lack of resources, and the fact that people were increasingly being deprived of choices that would facilitate decent living conditions (United Nations Development Programme [UNDP], 1998), as well as to the escalating denial of human beings’ right to fulfill their most basic needs (Yunus, 1994). According to Englama and Bamidele (1997), people suffering from poverty are unable to provide sufficiently for their themselves (e.g., clothing, decent accommodation, and food, Fallavier, 1998); cannot fulfill their social and economic responsibilities; cannot access beneficial employment and acquire the skills and resources needed to find suitable employment; and have insufficient access to economic and social infrastructures (e.g., health services, education, potable water, sanitation, and roads). Their economic opportunities are also often limited by, for instance, their parents’ financial situation, their race, and their religious beliefs (Solon, 1999).

The 21st century witnessed the introduction of a variety of definitions of poverty that are related to the economic perspective. They include defining poverty in terms of income (or prosperity) level below the generally accepted minimum (Atoyebi et al., 2008); in terms of a high unemployment rate, a low median income, and significant income disparity (Addae-Korankye, 2014); and in terms of socioeconomic instability, vulnerability, occupational uncertainty, and social exclusion (Weiss and Montgomery, 2005). Others have defined poverty as the deprivation of basic necessities, pleasures, possessions, and services (usually taken for granted) (Weiss and Montgomery, 2005); as food shortage or lack of shelter; as being ill without access to a doctor; as losing a child who fell ill through drinking contaminated water; as incapacity; and as lack of representation at social and political forums or not being free (World Bank, 2005). Lastly, the Swedish International Development Cooperation Agency [SIDA] (2005) defines poverty as the lack of money and money-making opportunities, while Corbett (2007) describes poverty as the lack of basic necessities (e.g., adequate, nutritious food, clothing, housing, clean water, and access to health services) needed to execute a job efficiently.

The Multidimensional Poverty Index (MPI) (Alkire et al., 2015) is an international measure of acute poverty used in over 100 developing countries. It complements and extends traditionally applied income-based poverty measures by including the deprivations that many people experience in, for instance, education (e.g., only a few years of schooling and minimal school attendance), health (e.g., high child mortality and inadequate nutrition), and living standards (e.g., a lack of cooking fuel, sanitary facilities, water, electricity, and proper surfaces or ceilings in homes). An assessment of poverty at the individual level can be used to create a comprehensive image of people living in poverty; permits comparisons both across and within countries, regions, and the world by ethnic group and location (urban vs. rural); and enables policy makers to obtain an overview of resources and devise strategic plans to deal with poverty (Alkire et al., 2015). Chakravarty et al. (2015) propose the poverty line as a way of assessing poverty by defining it on the basis of income position. Absolute position ranks individual income relative to an accepted (minimum) daily income in terms of its purchasing power parity for household consumption. Relative position ranks individual income in terms of a reference income (e.g., the mean or median income).

### The Sociological Perspective on Poverty

From a sociological perspective, poverty is defined as the inability to achieve a minimum standard of living – a state that is generally feared and viewed as extremely undesirable. Whatever citizens do should be aimed at preventing or eradicating poverty (World Bank, 1990). Ajuzie (2000) describes poverty as living without essential daily necessities such as adequate food, clothing, shelter, funding, and water supplies, thus accentuating inequality and social injustice, and Aworawo (2000) and Zakaria (2006) established links between poverty, loss of livelihood, inequality, and youth impatience. Okemakinde (2010) regards poverty as the inability to afford decent food, medical care, recreation, decent shelter and clothing, and to meet basic family needs and community obligations.

The sociological definition of poverty (Enciclopedia delle Science Sociali, 2015, Treccani) is that poverty has material features (e.g., lack of food and other essential goods), non-material features (e.g., disrespect and undermined dignity), and intergenerational features (poverty is generally transmitted from one generation to another). Anti-poverty policies should therefore be aimed at promoting self-realization by all members of society, especially the poorest people. The sociological definition of poverty indicates lack of social, political, and cultural goods and services (Enciclopedia delle Science Sociali, 2015, Treccani) and implies that such policies should help people suffering from poverty achieve a decent living standard by creating the socioeconomic conditions needed to grant them equal access to opportunities.

Adeola (2000) and Nwenearizi (2011) argue that poverty is a relative concept. Poor people lack material goods and also often have little confidence in others (Nwenearizi, 2011). The poor tend to live in inaccessible rural areas or in urban slums (Nwenearizi, 2011), and a link has also been established between poverty and minority status (e.g., black, Hispanic) and family structure, with unmarried or single people more likely to be poor (Cox and Healey, 2003).

Brady (2003) makes the following recommendations about what should be done about poverty: (1) a comparison should be drawn between different historical poverty and inequality trends, (2) poverty should be regarded as relative rather than absolute, (3) poverty should be conceptualized as and promotes social exclusion, (4) poverty indices should measure the profundity of inequality among the poor, and (5) taxes and state benefits (e.g., child grants) should be considered when family resources are calculated.

### The Psychological Perspective on Poverty

Psychological and sociological conceptualizations of poverty differed widely in the past and still do today (Di Fabio, 2015). From a sociological perspective, poverty was previously regarded as being shaped by economic, political, and structural factors (e.g., conditions of unemployment or differences in educational opportunities) (Lewis, 1968), and from a psychological perspective, poverty was regarded as being caused by individuals’ lack of effort and initiative and the result of character weakness (Allen, 1970). During the second half of the 20th century, psychology was largely unsuccessful in its attempts to broaden our understanding of poverty and to reduce poverty (Carr and Sloan, 2003). While the current sociological perspective on poverty takes into account the role of material deprivation as well as the lack of social, political, and cultural benefits and services (Kotecha et al., 2013), the current psychological perspective sees poverty in terms of people’s feelings of insecurity as well as their unmet needs, including unmet cognitive and affective psychological needs (Underlid, 2007).

During the last decade of the previous century, psychology contributed to poverty alleviation in three major life domains, namely health and welfare, social and organizational change, and personal and educational development (Carr and MacLachlan, 1998). American Psychological Association [APA] (2000) announced its intention to reduce poverty and highlighted the severe effect of poverty on people’s psychological well-being. However, psychologists have always found it difficult to conceptualize and define poverty satisfactorily because of the numerous causative factors and consequences, meaning that it cannot be studied as a unitary psychological construct in isolation (Brown and Lent, 2008).

Carr and Sloan (2003) argue that psychology’s interest in poverty revolves around its relatedness to powerlessness and injustice and the fundamental insecurities that it causes. Prilleltensky (2003), too, emphasizes poverty’s connection with powerlessness and believes psychology’s interest relates to its avowed aim to promote wellness, to resist exploitation, and to help people fulfill their basic needs.

Some authors define poverty from an attribution theory perspective (Kelley, 1967; Furnham, 2003). From an individualist point of view, poverty is brought about by the behavior of the poor. While the structural position attributes poverty to external social and economic factors, the fatalistic position attributes poverty to the role of fate. Underlid (2007) believes that the subjective meaning of poverty should be considered in addition to cognitive considerations. Whereas cognitive aspects include poor people’s experience of insecurity and their belief that significant others fail to support them, affective aspects of poverty relate to feelings of anxiety, panic, a devalued sense of self, anger, shame, guilt, sadness, and loss of autonomy. Poverty does not pertain only to an unwanted state experienced by most of the world’s population but also relates to deprivation as an unwelcome psychological state that is associated with a variety of life-expectancy limiting emotional and physical health issues (Anand and Lea, 2011). This means that behavioral economics (the study of the effects of psychological, social, cognitive, and emotional factors on the economic decisions of individuals and institutions) should be included in any discussion about poverty (Anand and Lea, 2011). Psychological insight into attitudes to debt and credit, for instance, could help indigent individuals improve their financial situation and thus help alleviate poverty.

From a psychological point of view, poverty may be caused or prevented by negative or positive individual as well as contextual factors. Individual factors that may help prevent or help people escape from poverty include the ability to understand the concepts of credit availability and debt management (Livingstone and Lunt, 1992; Anand and Lea, 2011), diligence, leadership skills, social skills, confidence, openness, emotional stability, energy, and a well-developed sense of self-esteem (Farkas, 2003), self-discipline, and money management skills (e.g., managing financial affairs meticulously and avoiding excessive spending) (Webley and Nyhus, 2001). Contextual factors that may help people avoid the poverty trap include a financially stable family background (Bowles and Gintis, 1976) as well as stable and financially sound consumption patterns of comparable reference groups (Lea et al., 1995; Schor, 1998).

Most importantly, poverty influences people’s choices negatively. Poor people have fewer choice options and often have a limited understanding of choices, which means that they are unlikely to choose appropriately from the choices available (Chakravarti, 2006). Likewise, poverty has a negative influence on people’s ability to adapt (Schmitt and Pilcher, 2004; Blustein et al., 2014; Osborne and Weiner, 2015; Thompson, 2015), on their ability to enjoy life and to avoid pain (Andrews and Withey, 1976; Kahneman et al., 1999), and on their ability to realize their potential and use their resources and strengths optimally (Vázquez et al., 2006; Ryff and Singer, 2008). Moreover, poverty limits people’s ability to assess the severity of threatening situations (Jacobson, 1991; Schneier, 2008), condemns them to membership of isolated groups with a similar status (Gylfason and Zoega, 2003; Blackford, 2006), and diminishes their quality of life and self-perceptions (in terms of, for example, their position in life in the context of their culture and value systems and with respect to their goals, expectations, standards, and concerns) (World Health Organization [WHO], 2002).

Psychology can contribute to poverty alleviation by (a) promoting psychosocial empowerment (e.g., by facilitating access to beneficial employment, formal education, and decent work; by promoting social equality, human rights, and by promoting engagement in decision-making and capacity-building networks) (Psychological Coalition at the United Nations [PCUN], 2012), mental health care and social protection, and psychosocial well-being (World Health Organization [WHO], 2002); (b) addressing the needs of marginalized and disenfranchised groups (those most at risk as regards poverty and related psychosocial and mental health problems); (c) evaluating the effectiveness of programs aimed at eradicating poverty; and (d) making calls to action to elicit an immediate response from established institutions and volunteers alike.

Regarding the development of psychological measures of attitudes toward poverty (widely regarded as a meaningful predictor of poverty), the *Attitude Towards Poverty* (*ATP*) scale (Gesinde et al., 2014) was developed to assess five major areas believed to perpetuate poverty, namely unemployment (lack of work), gambling (addiction to gambling), reckless spending (squandering money on unnecessary commodities), religion (some religions are less concerned about poverty reduction), and stealing (including robbery and theft). Used in a preventive framework for poverty reduction, this instrument can help identify effective poverty reduction strategies.

## Discussion

This article used a transdisciplinary interpretive lens to broaden reflections on decent work and poverty. Decent work and poverty were defined and elaborated on from philosophical, juridical, economic, sociological and psychological perspectives. Our aim was to propose a tentative transdisciplinary reflection on and comparison of the concepts of decent work and poverty. Below, we briefly summarize and compare the five perspectives referred to above.

According to Peruzzi (2015), the philosophical perspective on decent work focuses on dignity as intrinsic to decent work and emphasizes people’s right to be different to other people (in terms of life and work). The author foregrounds four aspects of decent work, namely freedom, equity, security, and dignity. From a juridical perspective, decent work is defined in terms of basic rights, such as the right to work and freedom to choose employment, to fair working conditions, to protection against unemployment, to fair remuneration, and to worthy existence and human dignity (United Nations, 1948; Faioli, 2009). The economic perspective on decent work emphasizes remuneration (fair wages and equal remuneration for the same work, a decent living for workers and their families, a safe and healthy occupational environment, equal promotion opportunities for all, and sufficient rest, leisure opportunities, and reasonable working hours and holidays) (United Nations Economic and Social Council, 2006). Three work-related profiles are identified: a basic profile (pertaining to daily working conditions in general), a relations profile (pertaining to social aspects of the occupation), and a fairness profile (with all people having access to decent work, Lavagnini and Mennella, 2015). The sociological perspective considers decent work in terms of “[a summary of] the aspirations of people in their working lives, opportunities for work that is productive and delivers a fair income, security in the workplace and social protection for families, better prospects for personal development and social integration, freedom for people to express their concerns, organize and participate in the decisions that affect their lives and equality of opportunity and treatment for all women and men” (International Labour Organization [ILO], 2015, n.p.). It also prohibits abusive occupational practices such as bonded labor. The psychological perspective emphasizes needs satisfaction (e.g., the need for power, relationships, and self-determination) (Blustein, 2006), the promotion of opportunities for progress, the prevention of marginalization, the reduction of work stress (Bell and Newitt, 2010; Carr et al., 2013), helping people gain insight into their potential (The International Association for Educational and Vocational Guidance [IAEVG], 2001; Athanasou, 2010; Di Fabio and Maree, 2013), the boosting of individual resources (Di Fabio and Kenny, 2012; Di Fabio and Palazzeschi, 2012; Di Fabio and Saklofske, 2014), the advancement of individuals, families, communities, and nations, and the encouragement of sustainable development (The International Association for Educational and Vocational Guidance [IAEVG], 2001; Di Fabio, 2015; Guichard and Di Fabio, 2015).

### Differences between Definitions of the Terms Decent Work and Poverty

Key differences have emerged between definitions of the terms decent work and poverty. Decent work refers explicitly to the right to work, freedom to choose employment, just and favorable working conditions, protection against unemployment, the right to equal pay for equal work, safe and wholesome working conditions, opportunities for adequate self-construction (realizing one’s talents and potential), gender and racial equality, guaranteeing workers’ rights, promoting social dialog related to decent work, self-determination, fair and sustainable development, health, and well-being. Poverty refers explicitly to deprivation, shortage, deficiency, indigence, paucity, privation, denial of human rights, social exclusion, stigmatization, powerlessness, anxiety, impaired sense of self or self-images, anger, shame, guilt, sadness, as well as loss of autonomy, and well-being.

### Similarities in Terms Used to Describe Perspectives on Decent Work and Poverty

Similarities in terms use to describe perspectives on the concepts decent work and poverty are indicated by the following keywords or terms: human dignity or absence thereof, a decent or non-decent living standard, insecurity versus security, unmet versus meeting of basic and psychological needs, power versus lack of power, justice versus lack of justice, denial versus promotion of human rights, equality versus inequality, freedom versus lack of freedom, employment versus lack of employment, social protection versus lack of social protection, livable income versus lack of livable income, marginalization, and a lack of versus promotion of well-being.

The five perspectives provide a good basis for a more expansive definition of decent work and poverty.

### Expanded Definition of Decent Work and Poverty

Considering what has been written above, it seems feasible to recommend that the following aspects be included in future definitions and operationalizations of decent work and poverty: Decent work helps all workers attain a sense of self-respect and dignity, experience freedom and security in the workplace, and (as far as possible) is afforded the opportunity to choose and execute productive, meaningful and fulfilling work that will enable them to construct themselves adequately and without restrictions and make social contributions. Moreover, the focus in discussions on the topic should emphasize the importance of preventing marginalization of employees, helping them find long-term employment, receive equal pay for equal work, and are protected by labor laws (which includes the right to join labor unions). The latter also includes meeting workers’ legitimate need for power, sound relationships in the workplace, acknowledgment of their work-related achievements, and authentic self-determination. Decent work ultimately aims to combat and alleviate poverty and precludes any and all forms of damage to workers.

We believe that this expanded definition could have important implications for the way in which researchers use these constructs (poverty and decent work) in their work and for interventions aimed at reducing poverty and promoting decent work. In the next section, we use this definition as a basis for discussing the quest for decent work and poverty alleviation.

### Acquiring Well-Being by Accessing Psychological and Tangible Opportunities to Improve Individual Growth and Development

Guichard and Di Fabio (2015) believe that acquiring well-being by accessing psychological and tangible opportunities to improve individual growth and development is intrinsically embedded in definitions of decent work and poverty. However, while the International Labour Organization [ILO]’s (1999, 2015) key definition of decent work underscores the importance of promoting well-being, Blustein (2008), Swanson (2012), Wanberg (2012), and Guichard (2013) state that dignity (an aspect of well-being) should be included in definitions of decent work. Poverty reduction, too, is closely linked to the promotion of the well-being. This view is supported by the philosophical perspective on poverty, which holds that poverty reduction through education and knowledge acquisition (generally regarded as prerequisites for democracy) can lead to people’s well-being (Peruzzi, 2009). The psychological perspective, more than any other perspective, links poverty reduction to well-being (American Psychological Association [APA], 2000), yet it has not adequately inspired meaningful contributions to poverty reduction in terms of promoting individual well-being (Carr and Sloan, 2003; Maree, 2015b).

Viewed from a decent work and poverty perspective, the promotion of well-being relates to the distinction introduced by positive psychology (Seligman and Csikszentmihalyi, 2000; Seligman, 2002) between hedonic well-being (pleasure attainment and pain avoidance) (Andrews and Withey, 1976; Kahneman et al., 1999) and eudaimonic well-being (functioning optimally and realizing one’s potential by exploiting one’s resources and strengths) (Vázquez et al., 2006; Ryff and Singer, 2008). While the first step to attaining decent work is ensuring that survival needs are met (through fair wages, safe working conditions, family security – all of which are connected to hedonic well-being), the second step is to conceptualize it in terms of the opportunity to construct oneself adequately and that invokes the importance of eudaimonic well-being (which provides a measure of workers’ well-being). People who give evidence of experiencing a sense of self-realization and meaning in work-related contexts inevitably give evidence of being more fully functioning than those who are not (Ryan and Deci, 2001). Work may thus be regarded as decent if it enables workers to obtain meaningfulness, fulfillment, self-realization (aspects of the authentic self) and facilitates purposeful identitarian awareness (Di Fabio, 2014b).

### Employing Intrapreneurial Self-Capital to Promote Decent Work and Combat Poverty

Individual psychological resources are a key means of escaping from poverty and promoting the attainment of decent work. Marginalization and economic constraints impede the attainment of decent work; however, drawing on psychological experiences and resources can moderate the effect of these limiting contextual factors and thus promote attainment of decent work and reduction of poverty. In addition to the value of possessing a proactive personality (Li et al., 2010), critical consciousness (Watts et al., 2011), perceived social support (Blustein, 2011), and work volition (the capacity to make occupational choices despite constraints; Duffy et al., 2012), a novel nucleus of individual resources, namely Intrapreneurial Self-Capital (ISC) (Di Fabio, 2014a) warrants mentioning here. Measured by the *Intrapreneurial Self-Capital Scale* (Di Fabio, 2014a), ISC can be developed through training. It comprises a nucleus of individual intrapreneurial resources that enable people to manage frequent changes and transitions by creating innovative solutions when confronted with environmentally imposed restrictions and turn restrictions into resources (Di Fabio, 2014a). A higher order construct, the ISC consists of seven specific constructs considered fundamental resources to contend with challenges posed by the 21st century occupational environment. These constructs are Core Self-Evaluation in terms of self-esteem, self-efficacy, locus of control, and the absence of pessimism (Judge et al., 2003); Hardiness in terms of commitment, control, challenge (Maddi, 1990); Creative Self-Efficacy (individuals’ perceptions about being able to face and solve problems creatively; Tierney and Farmer, 2002); Resilience (the perceived ability to cope with and continue to withstand adversity adaptively; Luthar et al., 2000) and to implement adaptive strategies to manage discomfort and adversity (Tugade and Fredrickson, 2004); Goal Mastery (the pursuit of unceasingly developing one’s own skills) (Midgley et al., 2000); Decisiveness (the perceived ability to make timeous decisions in all contexts) (Frost and Shows, 1993); and Vigilance (careful and adaptive examination of relevant information in decision-making processes) (Mann et al., 1997). There is empirical evidence that ISC scores correlate positively with scholastic success, employability, and career decision-making self-efficacy and negatively with career decision-making difficulties (Di Fabio, 2014a).

The ISC unlocks promising research perspectives and can be used to moderate between conditions of marginalization and economic constraints on the one hand and promote decent work and reduce poverty on the other hand. Moreover, a person’s ISC can be developed through targeted, tailored training (Di Fabio, 2014a). Guichard highlights the potential value of ISC in enabling marginalized people to collaborate in organizing systems of local exchange trading systems (LETS, Guichard, 2013). Maree (2015a) states that currently the aim of life design counseling for career construction, guidance, and life counseling intervention is to help people, individually and in groups, deal with transitions brought about by fundamental changes in occupational contexts. Guichard (2013, 2015) believes that ISC development can play an important role in achieving this aim. Furthermore, guidance and counseling intervention in the 21st century should be aimed primarily at helping people facing major challenges (young people without work, peripheral workers, people who have been unemployed for a long time, and so on). This aim can be achieved by promoting the development of LETS and associated systems that support the creation and organization of LETS. Career and life counselors are urged to help in this regard. Lastly, it should be noted that LETS and LETSpreneurial Self-Capital should be contextualized in terms of the community to which they belong, taking into account cross-cultural factors. The enhancement of intrapreneurial self-capital of individuals through targeted training can promote the development of personal resources that could enable them to emerge from conditions of exploitation, economic difficulties, the struggle to find decent work, and, eventually, combat poverty.

### Limitations of the Study

First, we are cognizant of the fact that some colleagues may regard the fact that we did not strictly adhere to the ‘standard’ (semi-quantitative) procedure to conduct a systematic qualitative overview of the literature as a major limitation of the study. Second, some of the documents that we studied were not peer-reviewed. Third, we are aware that other researchers, while analyzing the same sources, may arrive at different findings. Lastly, despite this being a collaborative, international study, we realize that our insights and findings are bound by time, space, and our idiosyncratic perspectives on matters.

## Conclusion

The 17 sustainable goals for global development as stated by the UN (Sustainable development goals, 2015) include:

(1) Ending poverty in all its forms everywhere.
(3) Ensuring healthy lives and promoting the well-being of all people.
(8) Promoting sustained, inclusive, and sustainable economic growth, full and productive employment, and decent work for all.

To achieve these three goals, it is more important to devise medium- and long-term projects to help victims of poverty not only survive but develop and grow than help people in emergency situations at a given point in time, which, on its own, will solve nothing and may even divert attention from the real aim, namely to help poor people escape from poverty and find decent work (Peruzzi, 2015). We recommend that decent work agendas should focus less on interventions at a tertiary prevention level and more on interventions at a secondary and, especially, a primary level of prevention (Hage et al., 2007; Kenny and Hage, 2009). This calls for a reprioritization of the levels of intervention and the re-allocation of available resources. Purposeful identitarian awareness needs to be instilled into helping agenda projects and actions. Interventions should be designed not only to respond to emergency situations but, especially, to break the vicious cycle of emergency occurring → resolving the emergency → another emergency occurring → resolving the emergency → another emergency occurring →. It is essential to identify the level of intervention needed to resolve particular challenges and to tailor different kinds of interventions to deal not only with the idiosyncratic challenges reactively but, more importantly, to devise strategies aimed at pro-active prevention of challenges because intervening at the level of primary prevention lies at the heart of any strategy. Radical intervention is needed to ensure that future generations not only survive but, more importantly, escape from poverty, develop, grow, and express themselves meaningfully through decent work.

The importance of timeous, pre-emptive, transnational intervention to ensure decent work for all workers and to alleviate poverty worldwide is clear. Rising unemployment across the globe and limited access to decent work have led to widespread poverty and feelings of profound frustration among many millions of people. This has played a key role in the global migration trends of the past few years, the growing socio-economic inequality, and the ever-widening gap between the rich and the poor. The future of humankind will be bleak indeed if we do not join hands today to overcome these challenges in a spirit of collaboration.

## Author Contributions

AD conceptualized the review, collected the materials and organized them. Then AD and JM wrote the paper together and read and revised the manuscript several times together.

## Conflict of Interest Statement

The authors declare that the research was conducted in the absence of any commercial or financial relationships that could be construed as a potential conflict of interest.

## References

[B1] Addae-Korankye A. (2014). Causes of poverty in Africa: a review of literature. *Am. Int. J. Soc. Sci.* 3 147–153.

[B2] Adeola F. (2000). Entrepreneurial skills, job creation, and poverty reduction in Nigeria. *Paper presented at the AIESEC Annual Alumni Conference* Lagos.

[B3] Agassi J. B. (1982). *Comparing the Work Attitudes of Men and Women.* Aldershot: Gower.

[B4] Ajuzie M. U. (2000). “The UBE and the factor of poverty,” in *Proceedings of the 4th National Conference of NAEND* (Okene: Federal College of Education).

[B5] Alkire S. Roche J. M. Seth S. Sumner A. (2015). Identifying the poorest people and groups: strategies using the Global Multidimensional Poverty Index. *J. Int. Dev.* 27 362–387. 10.1002/jid.3083

[B6] Allen V. L. (1970). Theoretical issues in poverty research. *J. Soc. Issues* 26 149–167. 10.1111/j.1540-4560.1970.tb01720.x

[B7] American Psychological Association [APA] (2000). *Resolution on Poverty and Socioeconomic Status.* Available at: http://www.apa.org/about/policy/poverty-resolution.aspx

[B8] Anand P. Lea S. (2011). The psychology and behavioral economics of poverty. *J. Econ. Psychol.* 32 284–293. 10.1016/j.joep.2011.01.001

[B9] Andrews F. M. Withey S. B. (1976). *Social Indicators of Well-Being: America’s Perception of Life Quality.* New York, NY: Plenum.

[B10] Arneson R. J. (2007). Shame, stigma, and disgust in the decent society. *J. Ethics* 11 31–63. 10.1007/s10892-006-9007-y

[B11] Athanasou J. A. (2010). Decent work and its implications for careers. *Aust. J. Career Dev.* 19 36–44. 10.1177/103841621001900108

[B12] Atoyebi T. A. Owoeye M. O. Adeoye A. M. A. (2008). Poverty and the attendant impacts on Nigerian rural women participation in polities: implication for sustainable democracy and good governance. *Hurage* 1 274–290.

[B13] Aworawo D. (2000). Ethnic crisis and political instability in Equatorial Guinea. *J. Cult. Stud.* 2 119–132. 10.4314/jcs.v2i1.6236

[B14] Bell S. Newitt K. (2010). *Decent Work and Poverty Eradication: Literature Review and Two-Country Study.* London: Ergon Associates.

[B15] Blackford R. (2006). *Genetic Enhancement and the Point of Social Equality.* Willington, CT: Institute for Ethics and Emerging Technologies.

[B16] Blustein D. L. (2006). *The Psychology of Working: A New Perspective for Career Development, Counseling, and Public Policy.* Mahwah, NJ: Erlbaum.

[B17] Blustein D. L. (2008). The role of work in psychological health and well-being: a conceptual, historical, and public policy perspective. *Am. Psychol.* 63 228–240. 10.1037/0003-066X.63.4.228 18473608

[B18] Blustein D. L. (2011). A relational theory of working. *J. Vocat. Behav.* 79 1–17. 10.1016/j.jvb.2010.10.004

[B19] Blustein D. L. (2013). “The psychology of working: a new perspective for a new era,” in *Oxford Handbooks of the Psychology of Working* ed. Blustein D. L. (New York, NY: Oxford University Press).

[B20] Blustein D. L. Kenny M. E. Kozan S. (2014). “Education and work as human birthrights: eradicating poverty through knowledge, innovation, and collaboration,” in *Barriers to and opportunities for poverty reduction: Prospects for private sector led interventions* ed. United Nations Development Program 38–62. Istanbul: UNDP Istanbul Center for Private Sector in Development.

[B21] Booth A. (2001). Cochrane or cock-eyed? how should we conduct systematic reviews of qualitative research? *Paper Presented at the Qualitative Evidence-Based Practice Conference, Taking a Critical Stance*. Coventry: Coventry University.

[B22] Bowles S. Gintis H. (1976). *Schooling in Capitalist America: Educational Reform and the Contradictions of Economic Life.* New York, NY: Basic Books.

[B23] Brady D. (2003). The politics of poverty: left political institutions, the welfare state, and poverty. *Soc. Forces* 82 557–588. 10.1353/sof.2004.0004

[B24] Brandt W. (1980). *North-South: A Programme for Survival.* Report of the Independent Commission on International Development Issues. Cambridge, MA: MIT Press 304

[B25] Braun V. Clarke V. (2006). Using thematic analysis in psychology. *Qual. Res. Psychol.* 3 77–101. 10.1191/1478088706qp063oa

[B26] Brown S. D. Lent R. W. (2008). *Handbook of Counseling Psychology* 4th Edn New York, NY: John Wiley.

[B27] Burchell B. Sehnbruch K. Piasna A. Agloni N. (2014). The quality of employment and decent work: definitions, methodologies, and ongoing debates. *Camb. J. Econ.* 38 459–477. 10.1093/cje/bet067

[B28] Burton W. C. (2007). *Burton’s Legal Thesaurus.* New York, NY: McGraw-Hill.

[B29] Carr S. C. MacLachlan M. (1998). Actors, observers, and attributions for third world poverty: contrasting perspectives from Malawi and Australia. *J. Soc. Psychol.* 138 189–202. 10.1080/00224549809600370

[B30] Carr S. C. Sloan T. S. (2003). *Poverty and Psychology: From Global Perspective to Local Practice.* New York, NY: Springer Science and Business Media.

[B31] Carr S. C. Thompson L. F. Reichman W. McWha I. Marai L. MacLachlan M. (2013). *Humanitarian Work Psychology: Concepts to Contributions. SIOP White Paper Series*. Bowling Green, OH: Society for Industrial and Organizational Psychology, Inc.

[B32] Chakravarti D. (2006). Voices unheard: the psychology of consumption in poverty and development. *J. Consum. Psychol.* 16 363–376. 10.1207/s15327663jcp1604_8

[B33] Chakravarty S. R. Chattopadhyay N. Nissanov Z. Silber J. (2015). *Reference Groups and the Poverty Line: An Axiomatic Approach with an Empirical Illustration (No. UNU-WIDER Research Paper WP2015/002).* Helsinki: United Nations University World Institute for Development Economics Research.

[B34] Corbett T. J. (2007). *Poverty. In Encarta Encyclopaedia. Microsoft^®^ Student 2008 (DVD).* Redmond, WA: Microsoft Corporation.

[B35] Cox A. Healey J. (2003). “The poverty reduction strategies of the development cooperation agencies in the 1990s: their record and what they need to do,” in *The New Poverty Strategies: What Have They Achieved? What Have They Learned?* eds Booth A. Mosley P. (Houndmills: Palgrave Macmillan) 21–43.

[B36] Despouy L. (1996). *The Realization of Economic, Social and Cultural Rights.* Final Report on Human Rights and Extreme Poverty. UN Doc. E/CN.4/Sub.2/1996/13.

[B37] Di Fabio A. (2014a). Intrapreneurial Self-Capital: a new construct for the 21st century. *J. Employ. Couns.* 51 98–111. 10.1002/j.2161-1920.2014.00045.x

[B38] Di Fabio A. (2014b). “The new purposeful identitarian awareness for the twenty-first century: valorize themselves in the Life Construction from youth to adulthood and late adulthood,” in *The Construction of the Identity in 21st Century: A Festschrift for Jean Guichard* eds Di Fabio A. Bernaud J.-L. (New York, NY: Nova Science Publishers) 157–168.

[B39] Di Fabio A. (2015). Poverty and decent work: widening the perspective with a transdisciplinary approach. *Paper Presented at the UNESCO Chair Conference in Florence 2015 How Can Career and Life Designing Interventions Contribute to a Fair and Sustainable Development and to the Implementation of Decent Work Over the World?* Florence: University of Florence.

[B40] Di Fabio A. Kenny M. E. (2012). Emotional intelligence and perceived social support among Italian high school students. *J. Career Dev.* 39 461–475. 10.1177/0894845311421005

[B41] Di Fabio A. Maree J. G. (2013). The effectiveness of the Career Interest Profile (CIP). *J. Employ. Couns.* 50 110–123. 10.1002/j.2161-1920.2013.00030.x

[B42] Di Fabio A. Palazzeschi L. (2012). Incremental variance of the core self-evaluation construct compared to fluid intelligence and personality traits in aspects of decision-making. *Pers. Individ. Differ.* 53 196–201. 10.1016/j.paid.2012.03.012

[B43] Di Fabio A. Saklofske D. H. (2014). Comparing ability and self-report trait emotional intelligence, fluid intelligence, and personality traits in career decision. *Pers. Individ. Differ.* 64 174–178. 10.1016/j.paid.2014.02.024

[B44] Dizionario di Economia e Finanza (2012). *Dizionario di Economia e Finanza [Dictionary of Economy and Finance].* Firenze: Treccani.

[B45] Duffy R. D. Blustein D. L. Diemer M. A. Autin K. L. (2016). The psychology of working theory. *J. Couns. Psychol.* 63 127–148. 10.1037/cou0000140 26937788

[B46] Duffy R. D. Diemer M. A. Jadidian A. (2012). The development and initial validation of the Work Volition Scale–Student Version. *Couns. Psychol.* 40 291–319. 10.1177/0011000011417147

[B47] Enciclopedia delle Science Sociali (2015). *Enciclopedia Delle Science Sociali [Encyclopedia of Social Sciences].* Firenze: Treccani.

[B48] Englama A. Bamidele A. (1997). “Measurement issues in poverty,” in *Poverty Alleviation in Nigeria, Selected Papers for the 1997 Annual Conference of the Nigerian Economics Society* (Ibadan: Nigerian Economics Society) 141–156.

[B49] Faioli M. (2009). *Decency at Work: Della Tensione del Lavoro Alla Dignità. Categorie Interculturali e Sapere Giuridico [Decency at Work: From Tension of Work to Dignity. Intercultural Categories and Juridical Knowledge].* Roma: La Nuova Cutura.

[B50] Fallavier P. (1998). *Developing Micro-Finance Institutions in VIETNAM.* Doctoral dissertation, University of British Columbia Vancouver, BC.

[B51] FAQs/Useful definitions (UN sources) (n.d.). *FAQs/Useful Definitions (UN Sources).* Available at: http://www.fao-ilo.org/fileadmin/user_upload/fao_ilo/pdf/FAQs/Definitions__2_.pdf

[B52] Farkas G. (2003). Cognitive skills and noncognitive traits and behaviors in stratification processes. *Annu. Rev. Sociol.* 29 541–562. 10.1146/annurev.soc.29.010202.100023

[B53] Ferrari L. E. (2009). Decent employment and subjectivity in the workplace: notes for an agenda of labor policies in citizenship terms. *Orientat. Soc.* 9 1–8.

[B54] Frost R. O. Shows D. L. (1993). The nature and measurement of compulsive indecisiveness. *Behav. Res. Ther.* 31 683–692. 10.1016/0005-7967(93)90121-A 8216169

[B55] Furnham A. (2003). “Poverty and wealth,” *Poverty and Psychology: From Global Perspective to Local Practice* eds Carr S. C. Sloan T. S. (New York, NY: Springer) 163–183.

[B56] Gaiha R. (1993). *Design of Poverty Alleviation Strategy in Rural Areas (No. 115).* Rome: Food and Agriculture Organization.

[B57] Gesinde A. M. Temitope A. A. David O. I. (2014). The development and validation of vignette-type instrument for measuring attitude toward poverty. *Proc. Soc. Behav. Sci.* 159 442–446. 10.1016/j.sbspro.2014.12.404

[B58] Guichard J. (2009). Self-constructing. *J. Vocat. Behav.* 75 251–258.

[B59] Guichard J. (2013). “Career guidance, education, and dialogues for a fair and sustainable human development,” in *Proceedings of the Inaugural Conference of the UNESCO chair of Lifelong Guidance and Counselling* (Wrocław: University of Wroclaw).

[B60] Guichard J. (2015). How can career and life designing interventions contribute to a fair and sustainable development and to the implementation of decent work over the world? *Paper Read at the UNESCO Chair on Lifelong Guidance and Counseling World Conference in Florence 2015* (Florence: University of Florence).

[B61] Guichard J. Di Fabio A. (2015). “How can career and life designing interventions contribute to a fair and sustainable development and to the implementation of decent work over the world?,” in *Invited Keynote at the UNESCO Chair on Lifelong Guidance and Counseling World Conference in Florence 2015* (Florence: University of Florence).

[B62] Gylfason T. Zoega G. (2003). Education, social equality and economic growth: a view of the landscape. *CESifo Econ. Stud.* 49 557–579. 10.1093/cesifo/49.4.557

[B63] Hage S. M. Romano J. L. Conyne R. K. Kenny M. Matthews C. Schwartz J. P. (2007). Best practice guidelines on prevention practice, research, training, and social advocacy for psychologists. *Couns. Psychol.* 35 493–566. 10.1177/0011000006291411

[B64] Higgens J. P. T. Green S. (eds) (2011). *Cochrane Handbook for Systematic Reviews of Interventions Version 5.1.0.* Available at: handbook.cochrane.org

[B65] Hulme D. McKay A. (2005). “Identifying and measuring chronic poverty: beyond monetary measures,” in *Chronic Poverty Research Centre Working Paper 30* (New Delhi: Indian Institute of Public Administration) 1–35.

[B66] International Labour Organization [ILO] (1999). “Report of the director-general: decent work,” in *Proceedings of the International Labour Conference 87th Session* Geneva.

[B67] International Labour Organization [ILO] (2015). *Decent Work and the 2030 Agenda for Sustainable Development.* Available at: http://www.ilo.org/global/topics/sdg-2030/lang--en/index.htm

[B68] Jacobson D. (1991). “The conceptual approach to job insecurity,” in *Job Insecurity: Coping with Jobs at Risk* eds Hartley J. Jacobson D. Klandermans B. (London: Sage) 23–39.

[B69] Judge T. A. Erez A. Bono J. E. Thoresen C. J. (2003). The core self-evaluations scale: development of a measure. *Pers. Psychol.* 56 303–331. 10.1111/j.1744-6570.2003.tb00152.x

[B70] Kahneman D. Diener E. Schwarz N. (eds) (1999). *Well-Being: Foundations of Hedonic Psychology.* New York, NY: Russell Sage Foundation.

[B71] Kelley H. H. (1967). “Attribution theory in social psychology,” in *Nebraska Symposium on Motivation* ed. Levine D. (Lincoln, NE: University of Nebraska Press).

[B72] Kenny M. E. Hage S. M. (2009). The next frontier: prevention as an instrument of social justice. *J. Prim. Prev.* 30 1–10. 10.1007/s10935-008-0163-7 19082727

[B73] Kokaz N. (2007). Poverty and global justice. *Ethics Int. Aff.* 21 317–336. 10.1111/j.1747-7093.2007.00102.x

[B74] Kotecha M. Arthur S. Coutinho S. (2013). *Understanding the Relationship Between Pensioner Poverty and Material Deprivation.* DWP Research Report London: The Department for Work and Pensions 827

[B75] Lavagnini M. Mennella A. (2015). *Decent Work in Italy: The Basic-Relations-Fairness Proposal: Forum for Social Economics (No. ahead-of-print).* London: Routledge 1–20.

[B76] Lea S. E. Webley P. Walker C. M. (1995). Psychological factors in consumer debt: money management, economic socialization, and credit use. *J. Econ. Psychol.* 16 681–701. 10.1016/0167-4870(95)00013-4

[B77] Lewis O. (1968). *La Vida [The Life].* London: Panther.

[B78] Li N. Liang J. Crant J. M. (2010). The role of proactive personality in job satisfaction and organizational citizenship behavior: a relational perspective. *J. Appl. Psychol.* 95 395–404. 10.1037/a0018079 20230079

[B79] Livingstone S. M. Lunt P. K. (1992). Predicting personal debt and debt repayment: psychological, social and economic determinants. *J. Econ. Psychol.* 13 111–134. 10.1016/0167-4870(92)90055-C

[B80] Luthar S. S. Cicchetti D. Becker B. (2000). The construct of resilience: a critical evaluation and guidelines for future work. *Child Dev.* 71 543–562. 10.1111/1467-8624.00164 10953923 PMC1885202

[B81] Maddi S. R. (1990). “Issues and interventions in stress mastery,” in *Personality and Disease* ed. Friedman H. S. (New York, NY: Wiley) 121–154.

[B82] Mann L. Burnett P. Radford M. Ford S. (1997). The Melbourne Decision Making Questionnaire: an instrument for measuring patterns for coping with decisional conflict. *J. Behav. Decis. Mak.* 10 1–19. 10.1002/(SICI)1099-0771(199703)10:1%3C1::AID-BDM242%3E3.0.CO;2-X

[B83] Maree J. G. (2015a). Career Construction Counseling: a thematic analysis of outcomes for four clients. *J. Vocat. Behav.* 86 1–9. 10.1016/j.jvb.2014.10.001

[B84] Maree J. G. (2015b). Career construction counseling with a mid-career black male. *Career Dev. Q.* 64 20–35. 10.1002/cdq.12038

[B85] Midgley C. Maehr M. L. Hruda L. Z. Anderman E. Anderman L. Freeman K. E. (2000). *Manual for the Patterns of Adaptive Learning Scales.* Available at: http://www.umich.edu/~{}pals/PALS%202000V13Word97.pdf

[B86] Narveson J. (2004). Welfare and wealth, poverty and justice in today’s world. *J. Ethics* 8 305–348. 10.1007/s10892-004-4895-1

[B87] Nussbaum M. C. (2004). *Hiding from Humanity: Disgust, Shame, and the Law.* Princeton, NY: Princeton University Press.

[B88] Nwenearizi F. O. (2011). Strategies for poverty alleviation in rural area for a sustainable economic development in Nigeria. *J. Educ. Health Technol. Res.* 1 210–216.

[B89] Okemakinde T. (2010). Illiteracy and poverty in Nigeria: universal basic education as a panacea. *J. Educ. Appl. Psychol.* 3 143–158.

[B90] Osborne D. Weiner B. (2015). A latent profile analysis of attributions for poverty: identifying response patterns underlying people’s willingness to help the poor. *Pers. Individ. Differ.* 85 149–154. 10.1016/j.paid.2015.05.007

[B91] Osia K. (2010). *Beyond Juridical Abstraction: Poverty in a World of Plenty.* Available at: http://forumonpublicpolicy.com/vol2010.no4/archive.vol2010.no4/osia.pdf

[B92] Paul K. I. Moser K. (2009). Unemployment impairs mental health: meta-analyses. *J. Vocat. Behav.* 74 264–282. 10.1016/j.jvb.2009.01.001

[B93] Peruzzi A. (2009). *Scienza per la Democrazia [Science for Democracy].* Pisa: Edizioni Ets.

[B94] Peruzzi A. (2015). *Un Appello per la Dignità [A Plea for Dignity]. Counseling. Giornale Italiano di Ricerca e Applicazioni.* Available at: http://rivistedigitali.erickson.it/counseling/archivio/vol-8-n-2/

[B95] Prilleltensky I. (2003). Understanding, resisting, and overcoming oppression: toward psychopolitical validity. *Am. J. Community Psychol.* 31 195–201. 10.1023/A:1023043108210 12741700

[B96] Psychological Coalition at the United Nations [PCUN] (2012). *Psychological Contributions to the Eradication of Poverty.* Available at: http://www.worldpsyche.org/contents/14139/psychological-contributions-to-the-eradication-of-poverty

[B97] Ryan R. M. Deci E. L. (2001). On happiness and human potentials: a review of research on hedonic and eudaimonic well-being. *Annu. Rev. Psychol.* 52 141–166. 10.1146/annurev.psych.52.1.141 11148302

[B98] Ryff C. D. Singer B. H. (2008). Know thyself and become what you are: a eudaimonic approach to psychological well-being. *J. Happiness Stud.* 9 13–39. 10.1007/s10902-006-9019-0

[B99] Schmitt D. P. Pilcher J. J. (2004). Evaluating evidence of psychological adaptation: how do we know one when we see one? *Psychol. Sci.* 15 643–649. 10.1111/j.0956-7976.2004.00734.x 15447633

[B100] Schneier B. (2008). *The Psychology of Security.* Available at: https://www.schneier.com/essays/archives/2008/01/thepsychologyofse.html

[B101] Schor J. B. (1998). *The Overspent American.* New York, NY: Basic Books.

[B102] Seligman M. E. Csikszentmihalyi M. (2000). *Positive Psychology: An Introduction.* Washington, DC: American Psychological Association. 10.1037//0003-066x.55.1.5 11392865

[B103] Seligman M. E. P. (2002). “Positive psychology, positive prevention, and positive therapy,” in *Handbook of Positive Psychology* eds Snyder C. R. Lopez S. J. (New York, NY: Oxford University Press) 3–9.

[B104] Sen A. (1999). *Development as Freedom.* Oxford: Oxford University Press.

[B105] Solon G. (1999). “Intergenerational mobility in the labor market,” *Handbook of Labor Economics* Vol. 3 eds Orley A. Ashenfelter C. Card D. (Amsterdam: North-Holland) 1761–1800.

[B106] Somavia J. (1999). *The Decent Work Agenda.* Available at: http://www. molsmed.gov.tt/Portals/0/Decent%20Work%20Agenda.pdf 12296135

[B107] Stiglitz J. E. Sen A. Fitoussi J. P. (2010). *Report by the Commission on the Measurement of Economic Performance and Social Progress.* Paris: Commission on the Measurement of Economic Performance and Social Progress.

[B108] Sustainable development goals (2015). *17 Goals to Transform Our World.* Available at: http://www.un.org/sustainabledevelopment/development-agenda/

[B109] Swanson J. L. (2012). “Work and psychological health,” in *APA Handbook of Counseling Psychology* eds Fouad N. A. Carter J. A. Subich L. M. (Washington, DC: American Psychological Association) 3–27.

[B110] Swedish International Development Cooperation Agency [SIDA] (2005). *Health is Wealth.* Stockholm: SIDA.

[B111] The International Association for Educational and Vocational Guidance [IAEVG] (2001). *The Paris 2001 IAEVG Declaration on Educational and Vocational Guidance.* Available at: http://iaevg.net/iaevg.org/IAEVG/nav3937.html?lang=2andmenu=1andsubmenu=4

[B112] Thompson M. (2015). Poverty: third wave behavioral approaches. *Curr. Opin. Psychol.* 2 102–106. 10.1016/j.copsyc.2014.11.010

[B113] Tierney P. Farmer S. M. (2002). Creative self-efficacy: its potential antecedents and relationship to creative performance. *Acad. Manage. J.* 45 1137–1148. 10.2307/3069429

[B114] Treccani Etymological Dictionary (2012). *Dizionario Etimologico Treccani [Treccani Etymological Dictionary].* Firenze: Treccani.

[B115] Tugade M. M. Fredrickson B. L. (2004). Resilient individuals use positive emotion to bounce back rom negative emotional experiences. *J. Pers. Soc. Psychol.* 86 320–333. 10.1037/0022-3514.86.2.320 14769087 PMC3132556

[B116] Underlid K. (2007). Poverty and experiences of insecurity. A qualitative interview study of 25 long-standing recipients of social security. *Int. J. Soc. Welf.* 16 65–74. 10.1111/j.1468-2397.2006.00423.x 15842418

[B117] UNESCO (2009). *Poverty and Human Rights: UNESCO’s Antipoverty Projects.* Available at: http://portal.unesco.org/shs/en/ev.php-URL_ID=4666andURL_DO=DO_TOPICandURL_SECTION=201.html

[B118] United Nations (1948). *Universal Declaration of Human Rights.* Available at: http://www.un.org/en/documents/udhr/

[B119] United Nations [UN] (2003). *Searching for Innovations in Governance and Public Administration for Poverty Reduction: Concepts, Experiences and Lessons for the Future.* New York, NY: United Nations.

[B120] United Nations [UN] (2006). *Full and Productive Employment and Decent Work.* New York, NY: United Nations.

[B121] United Nations Development Programme [UNDP] (1998). *Human Development Report 1998.* New York: United Nations Development Programme. Available at: http://hdr.undp.org/sites/default/files/reports/259/hdr_1998_en_complete_nostats.pdf

[B122] United Nations Development Program [UNDP] (2004). *Human Development Report 2004: Cultural Liberty in Today‘s Diverse World.* Oxford: Oxford University Press.

[B123] United Nations Economic and Social Council (2006). *The Right to Work. General Comment No. 18.* New York, NY: United Nations Economic and Social Council.

[B124] Vázquez C. Hervás G. Ho S. (2006). Intervenciones clínicas basadas en la psicología positiva: fundamentos y aplicaciones. *Psicol. Conductual* 14 401–432.

[B125] Wanberg C. R. (2012). The individual experience of unemployment. *Annu. Rev. Psychol.* 63 369–396. 10.1146/annurev-psych-120710-100500 21721936

[B126] Watts R. J. Diemer M. A. Voight A. M. (2011). “Critical consciousness: current status and future directions,” in *Youth Civic Development: Work at the Cutting Edge. New Directions for Child and Adolescent Development* Vol. 134 eds Flanagan C. A. Christens B. D. (New York, NY: Wiley) 43–57. 10.1002/cd.310 22147600

[B127] Webley P. Nyhus E. K. (2001). Life-cycle and dispositional routes into problem debt. *Br. J. Psychol.* 92 423–446. 10.1348/000712601162275 11534738

[B128] Weiss J. Montgomery H. (2005). Great expectations: microfinance and poverty reduction in Asia and Latin America. *Oxf. Dev. Stud.* 33 391–416. 10.1080/13600810500199210

[B129] Wilson W. J. (1996). *When Work Disappears: The World of the New Urban Poor.* New York, NY: Knopf.

[B130] World Bank (1990). *World Development Report.* New York, NY: Oxford University Press.

[B131] World Bank (2005). *World Development Indicators 2005.* New York, NY: Oxford University Press.

[B132] World Health Organization [WHO] (2002). *The World Health Report 2002: Reducing Risks, Promoting Healthy Life.* Geneva: World Health Organization.

[B133] Yunus M. (1994). *Bunking on the Poor.* Dhaka: Grameen Bank.

[B134] Zakaria Y. (2006). *Youth, Conflict, Security, and Development. The Reality of Aid 2006.* Available at: http://www.realityofaid.org/wp-content/uploads/2013/02/RoA-Report-2006-Part-II.pdf

